# A higher burden of post-stroke depression and anxiety and their predictors among stroke survivors in the Amhara Regional State, Ethiopia, in 2024: a prospective multicenter study

**DOI:** 10.3389/fpsyt.2025.1545807

**Published:** 2025-04-22

**Authors:** Biruk Lelisa Eticha, Ermias Solomon Yalew, Destaw Marie Merawie, Samuel Teferi Chanie, Kaleb Assegid Demissie, Biruktawit Lelisa Eticha

**Affiliations:** ^1^ Department of Optometry, College of Medicine and Health Sciences, Comprehensive Specialized Hospital, University of Gondar, Gondar, Ethiopia; ^2^ Department of Physiotherapy, College of Medicine and Health Sciences, Comprehensive Specialized Hospital, University of Gondar, Gondar, Ethiopia; ^3^ Department of Health Systems and Policy, Institute of Public Health, College of Medicine and Health Sciences, University of Gondar, Gondar, Ethiopia; ^4^ Department of Health Informatics, College of Medicine and Health Science, Wachemo University, Hossana, Ethiopia

**Keywords:** depression, anxiety, stroke, Amhara Regional State, Ethiopia

## Abstract

**Background:**

A substantial proportion of stroke survivors suffer from post-stroke depression and anxiety. These mental disorders are linked to several modifiable risk factors and lead to severe functional impairment or premature death. There is a lack of evaluation, prevention, and treatment of these prevalent mental illnesses. This study aimed to investigate the overall burden of post-stroke depression and anxiety and their predictors in the Amhara Regional State, Ethiopia, in 2024.

**Methods:**

From 01 February to 01 April 2024, a multicenter cross-sectional study was conducted on 404 stroke survivors from five comprehensive specialized hospitals in the Amhara Regional State, Ethiopia. Five trained physiotherapists conducted interviews, reviewed medical records, and took physical measurements using a pretested, semi-structured questionnaire to obtain high-quality data for analysis. Descriptive statistics were taken into consideration to provide a broad overview of the data and distribution of conditions. Additionally, binary logistic regression was used to find predictors with a p-value of less than 0.2 that could be subjected to multivariate logistic regression analysis, which was used to find the significant associated factors. A p-value of less than 0.05 with a 95% confidence interval (CI) was deemed significant.

**Results:**

The prevalence of post-stroke depression and anxiety among stroke survivors in the Amhara Regional State was 64.1% (95% CI: 59.3–68.6%) and 45.5% (95% CI: 40.7–50.4), respectively. Variables including male sex [adjusted odds ratio (AOR)=1.97, 95% CI: 1.06–3.67], stroke complication presence (AOR=2.83, 95% CI: 1.64-4.88), and comorbidity presence (AOR=6.23, 95% CI: 3.91–9.19) were significantly associated with post-stroke depression. Retirement (AOR=1.64, 95% CI: 1.91–4.72), less time for hospitalization (AOR=2.05, 95% CI: 1.09–3.84), and comorbidity presence (AOR=2.09, 95% CI: 1.32–3.29) were the significantly associated factors of post-stroke anxiety.

**Conclusions:**

Relatively higher burdens of post-stroke depression and anxiety were observed among stroke survivors in the Amhara Regional State, Ethiopia. Variables such as sex, stroke complication, and comorbidity, and retirement, time for hospitalization, and comorbidity were predictors significantly associated with post-stroke depression and anxiety, respectively. Early mental health screening and diagnosis of old age, complicated case patients, and retired stroke survivors are required for early-stage interventions.

## Introduction

Stroke, a sudden neurologic deficit caused by acute cerebrovascular, ischemic, or hemorrhagic focal damage, is the second leading attributable cause of death in the world, with a prevalence of 101 million cases (1–3). In Ethiopia, stroke is the most common neurological disorder of patients admitted to general hospitals, and it has been reported as a major cause of mortality and morbidity (4).

The majority of stroke patients survive and become more likely to experience long-term physical, cognitive, and psychological effects. This condition forces them to live with a condition that costs the world economy $721 billion USD (0.66% of the global gross domestic product) (1, 5).

Depression and anxiety, as common types of mental disorders, are the leading mental disorders, contributing to approximately 14% of the total global burden of disease (6–8). These two conditions create significant social, behavioral, and economic burdens and are considered public health concerns (6). Accordingly, the presence of these mental disorders threatens the wellbeing of stroke survivors by elongating the time returning to work (9) and functional recovery (10–12), compromising cognitive health (13) and quality of life (14–16), and raising the mortality rate (17, 18). It has been observed that post-stroke depression (PSD) raised the healthcare cost of stroke survivors by 54%–63% (19, 20). The additional poor prognosis of stroke with increased mental healthcare expense resulting in extended hospitalization time could increase the cost to the patient (21).

Multifaceted worldwide studies estimated the prevalence of PSD and post-stroke anxiety (PSA) to be 14% (22) to 90% (23) and 15.7% (1) to 45.9% (24), respectively. In Ethiopia, no study conducted on PSA has been conducted and the PSD prevalence ranged from 27.5% (25) to 49.6% (26).

Despite PSD and PSA being observed in a significant proportion of stroke survivors and being associated with multiple modifiable risk factors (hypertension, heart disease, smoking, hypercholesterolemia, and unhealthy lifestyle) causing severe functional impairment and death, limited assessment, prevention, and management of these common mental disorders have been observed (27–29). A study revealed that an estimated 50% to 80% of the actual depression of stroke survivors was left undiagnosed by non-psychiatry professionals, while the real burden of the common mental disorders is yet to be elucidated (4).

Since quality and comprehensive data are vital for efficient, evidence-based, and applicable interventions, this study was planned to produce novel data addressing unexplored potential predictors, with a considerably large sample size and a wide area. This exclusive study aimed to investigate the burden of PSD and PSA and their predictors among stroke survivors admitted to comprehensive specialized hospitals (CSHs) in the Amhara Regional State, Ethiopia, in 2024.

## Materials and methods

### Study design, area, and period

This multicenter cross-sectional study was conducted in five CSHs in the Amhara Regional State from 01 February to 01 April 2024. These hospitals, namely, University of Gondar CSH, Debre Birhan CSH, Felege Hiwot CSH, Tibebe Ghion CSH, and Dessie CSH, are among the seven CHSs that provide comparable active physiotherapy and rehabilitation services in Ethiopia’s second most populous regional state for the vast territory and a diversified population, including the neighboring regions such as Benishangul Gumuz, Afar, Tigray, and Oromia regional states, providing a chance to be selected as an eligible study setting.

### Source population

All stroke survivors attending the physiotherapy outpatient clinics of the selected CSHs in the Amhara Regional State, Ethiopia.

### Study population

All stroke survivors who were attending and accessible at the physiotherapy outpatient clinics of selected CSHs at the time of data collection were included in the study population.

### Eligibility criteria

Individuals suffering from chronic and advanced-stage neurological disorders, such as spinal cord injury, traumatic brain injury, and brain space-occupying lesions, that hindered quality data collection were excluded. This spared stroke survivors who were in critical health conditions from additional burdens related to data collection. This was assessed by direct observation of the supervisor and chart review at the early beginning of the study subject selection process. Due to the special intensive clinical examinations and care required for non-adult patients, individuals aged 18 years and below were also excluded from the study. This was to avoid bias and ethical violations.

In addition, for the sake of consistent and quality data collection, individuals with diagnosed speech difficulties were excluded from the study.

### Sample size determination

The sample size was determined using a single population proportion formula. From research conducted in Ethiopia (26) and Nigeria (30), proportions of 0.496 and 0.197 for PSD and PSA among adult stroke survivors were used, respectively. Furthermore, the sample size calculation considered a 5% significance level for both formulas, and 5% and 4% margins of error were considered for depression and anxiety, respectively.


$$n_{\left(Depression\right)}\;=\frac{{\left({\text{Z}}_{\text{α}/2}\right)}^{2}*\text{P}\left(1-\text{P}\right)}{{\text{d}}^{2}}=\frac{{\left(1.96\right)}^{2}*0.496\left(1-0.496\right)}{0.05^{2}}=384$$

$$n_{\left(Anxiety\right)}\;=\frac{{\left({\text{Z}}_{\text{α}/2}\right)}^{2}*\text{P}\left(1-\text{P}\right)}{{\text{d}}^{2}}=\frac{{\left(1.96\right)}^{2}*0.197\left(1-0.197\right)}{0.04^{2}}=380$$

where n = sample size, Z = Z statistics for a 95% level of confidence, P = proportion of depression and anxiety [0.496 (31) and 0.197 (30), respectively], and d = margin of error.

After considering the non-response rate, the final sample sizes for PSD and PSA were 422 and 418, respectively. Because the sample size calculated for PSD was higher than that of PSA, we decided to use the larger one (422) as the final sample size to assess the burden of depression and anxiety and their predictors among stroke survivors in the Amhara Regional State.

### Sampling technique and procedure

**Figure 1:**
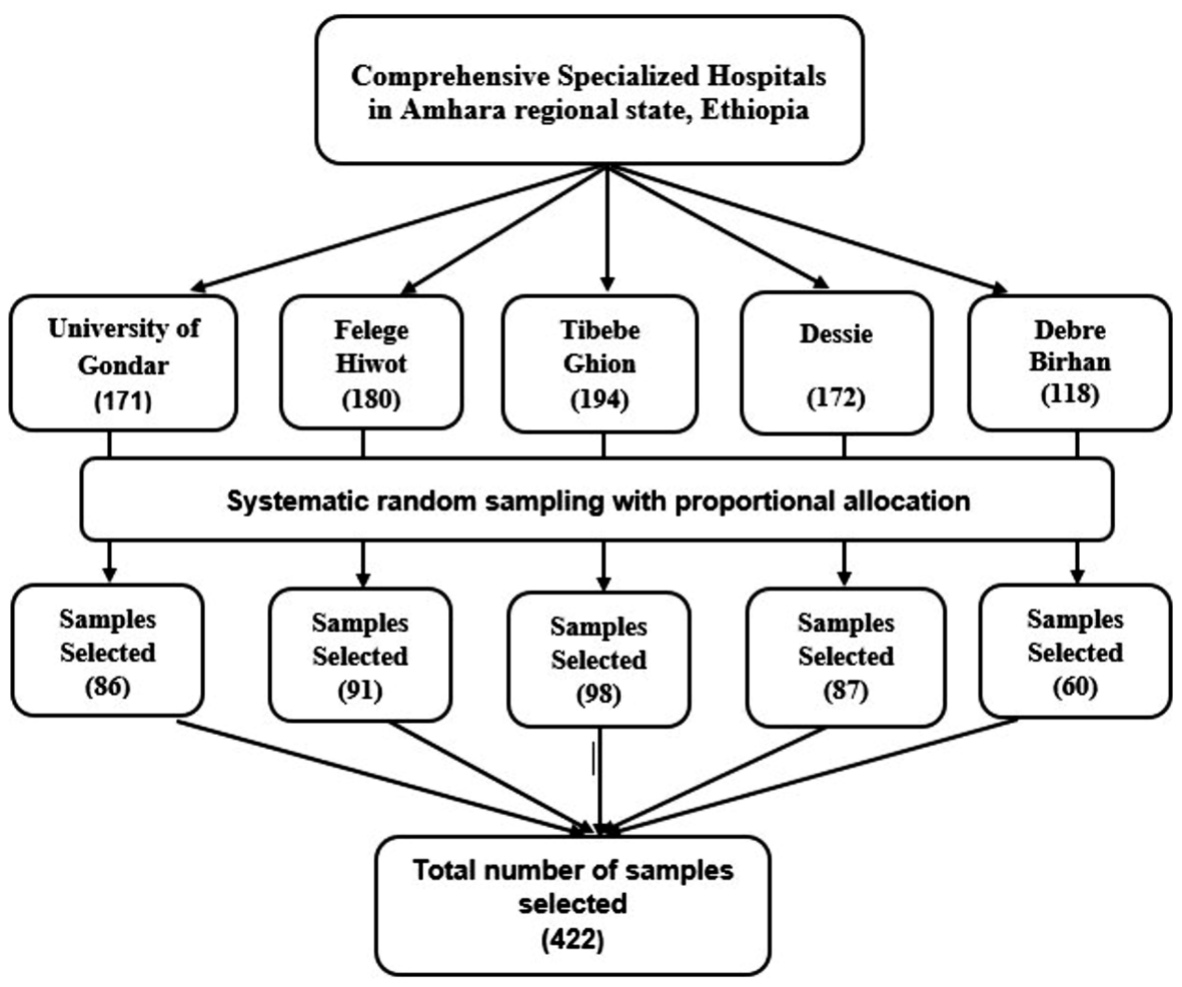
Schematic presentation of sampling technique and procedures used to assess the burden of post-stroke depression and anxiety and their predictors among stroke survivors in the Amhara Regional State, Ethiopia.

The information desk of each CSH unveiled that about 835 stroke survivors have been getting service in the physiotherapy clinics over a two-month period on average. Using a systematic random sampling technique, the study participants were chosen proportionately based on the number of stroke survivors obtaining service. The sampling interval (K) was calculated to be approximately two ( $\frac{N}{n}\;=\frac{835}{422}\;=\;2$ ). Following the lottery method selection of the first sample from the first two patients, data collection was carried out daily at K-intervals. (**Figure 1**).

#### Variables

##### Dependent variables

PSD and PSA

##### Independent variables

###### Socio-demographic and behavioral variables

Age, sex, residence, marital status, educational status, occupation, average monthly income, and cigarette smoking.

###### Clinical variables

Comorbidity, number of falls, time for hospitalization, type of stroke, duration of stroke, stroke complication, side of paralysis, duration of hospitalization, current medication, loss of consciousness, and body mass index (BMI).

#### Operational definitions

##### Depression

A total depression subscale score of ≥ 10 points out of a possible 21 points in the Patient Health Questionnaire-9 (PHQ-9) was used to diagnose clinically significant depression (32).

##### Anxiety

A total subscale score of ≥ 8 points out of a possible 21 points in the Hospital Anxiety and Depression Score (HADS) anxiety subscale was considered clinically significant anxiety (33).

##### Body mass index

The objectively measured weight in kilograms was divided by the height in meters squared to determine the BMI and then the calculated BMI was categorized as Underweight (the BMI< 18.50 kg/m^2^), Normal (the BMI 18.50 - 24.99 kg/m^2^), Overweight (the BMI 25.00 - 29.99 kg/m^2^) or obese (the BMI ≥30 kg/m^2^) (34).

##### Post-stroke fall

Any fall regardless of cause that was identified and reported by staff/patient/caregiver or documented in a medical recording chart was defined as a fall (35).

##### Urinary incontinence

The presence of urinary incontinence was defined as one or more wettings within a day after admission to the specific clinic they attended (36).

##### Cigarette smoking

Daily or occasional smoking of at least one stick of cigarette per day was deemed as cigarette smoking (37).

##### Comorbidity

A stroke survivor living with one or more systemic diseases, such as hypertension, diabetes mellitus, cancer, and myocardial disorders, was defined as comorbidity.

### Data collection tool and procedures

Five licensed MSc-holding physiotherapists who had at least 5 years of experience in diagnosis, care, and management of neurological conditions in clinics collected the data. Moreover, the data were gathered using an interviewer-administered, structured, pretested questionnaire composed of sociodemographic, behavioral, and clinical components. After the questionnaire was prepared by constructing questions that ultimately aimed to answer the raised study questions, it was administered using electronic devices using Kobo Toolbox version 2022.4.4 for data collection.

The study participants’ weight and height measurements were conducted using a standard weighing scale tool and tape measures, respectively. The physiotherapist data collectors then assessed and extracted the sociodemographic, behavioral, and clinical characteristics of the stroke survivors using an interview and physical examination. The final step was assessing the PSD and PSA status using the Amharic version of the PHQ-9 and HADS-14 questionnaires, respectively. The tools are highly reliable and used for screening, diagnosing, and monitoring the corresponding sensitive mental disorders (38).

The PHQ-9 is composed of nine items with a 4-item Likert scale from 0 to 3 (maximum score of 27 points). A total score of 10 and above was considered the cutoff point for PSD diagnosis (39). HADS has been found to be a reliable instrument for detecting states of anxiety in the setting of hospital outpatient clinics. Like PHQ-9, the HADS questionnaire used for anxiety assessment has seven items, with each item measured with a 4-point Likert scale ranging from 0 to 3 (40). The scores (higher scores denoting higher anxiety) of each subscale were summed separately. A total sub-scale score of ≥ 8 points out of a possible 21 denotes considerable anxiety (40). The validity and reliability of these tools in Amharic language, chronic patients, and comparable setups and socioeconomic conditions make these tools preferable over others (26). Additionally, the pretest confirmed the reliability of PHQ-9 and HADS-14 with Cronbach’s alpha values of 0.993 and 0.783, respectively.

### Data quality assurance

All of the data collection processes conducted by five trained clinical physiotherapists were conducted under the supervision of supervisors who were allocated to each of the study areas selected. The training given to data collectors was held for a day and focused on the technical and professional approaches to data collection. After the tool was prepared in Amharic, it was translated into English and retranslated back to Amharic by local language experts. Furthermore, to check the accuracy of responses, language clarity, consistency, and appropriateness, the tool was pretested on 5% of the sample size at St. Paul Hospital Millennium Medical College. Finally, immediately before analysis, the data underwent a checkup of outliers, missing values, and errors.

### Statistical analysis

Once the data were collected using Kobo Toolbox version 2022.4.4, it was exported to Stata version 14 for cleaning and analysis. The same statistical package was used to calculate descriptive statistics, such as proportion, frequency, and ratios, and analytic statistics. Considering a p-value of less than 0.2, eligible variables were selected and underwent multivariate binary logistic regression analysis. The final multivariate binary logistic regression model identified the final significantly associated variables using the cutoff point of a p-value less than 0.05 with a 95% confidence interval (CI). The model fitness, checked using the Hosmer and Lemeshow goodness of fit test, confirmed good model fitness (0.67 and 0.53 p-values for depression and anxiety, respectively).

### Ethical considerations

At the beginning of the investigation, ethical clearance (reference number ‘SOM/234/2024’) was obtained from the School of Medicine Ethical Review Board at the University of Gondar. Subsequently, official permission was acquired from the medical directors of all the selected CSHs. Regarding the data collection, all of the study subjects had the chance to provide their verbal informed consent, preceded by a thorough explanation of the aim, benefits, and risks of the study. They were also aware of their right to refuse to participate and give up their involvement at any point in the process. Furthermore, the privacy of the study participants was secured by classifying the questionnaire and eliminating any of the potential identifiers from any of the documents. Throughout the investigation, all the processes were conducted in accordance with the World Medical Association’s Declaration of Helsinki.

## Results

### Sociodemographic and behavioral characteristics of the study subjects

With a 95.7% response rate, 404 stroke survivors were involved in this study. The median age of the study subjects was 58 years, with a range of 20 to 90 years. Approximately three-fourths, 298 (73.8%), of the participants were living in urban areas, and above half, 205 (50.7%), of them were male (**Table 1**).

**Table 1:** Sociodemographic and behavioral characteristics of stroke survivors in the Amhara Region, Ethiopia (n=404).

Variable	Category	Frequency	Percentage
Age (in years)			
	20–39	36	8.9%
	40–64	200	49.5%
	> 65	168	41.6%
Sex			
	Male	205	50.7%
	Female	199	49.3%
Residence			
	Urban	298	73.8%
	Rural	106	26.2%
Marital status			
	Never married	45	11.2%
	Married	266	65.8%
	Separated	33	8.2%
	Divorced	11	2.7%
	Widowed	49	12.1%
Educational status			
	No formal education	120	29.7%
	Primary education	99	24.5%
	Secondary education	100	24.8%
	College and above	85	21.0%
Occupation			
	Farmer	41	10.2%
	Civil servant	57	14.1%
	Housewife	109	16.9%
	Merchant	76	18.8%
	Retired	29	7.2%
	Private	81	20.1%
	Other	11	2.7%
Average monthly income (ETB)			
	100–2,000	106	26.2%
	2,001–5,000	128	31.7%
	5,001–10,000	97	24.0%
	10,001–40,000	73	18.1%
Cigarette smoking			
	Yes	123	30.5%
	No	281	69.5%

n, sample size; ETB, Ethiopian birr.

### Clinical characteristics of the study subjects

Three-fourths, 307 (76.0%), of the study subjects visited the hospital within a day after the onset of stroke. Furthermore, the majority, 246 (60.9%) and 237 (58.7%), of the stroke survivors who participated in the study were suffering from the ischemic type of stroke and developed complications from stroke, respectively. Moreover, approximately two-thirds, 257 (63.6%), of the study participants had other confirmed systemic comorbidities (**Table 2**).

**Table 2:** Clinical characteristics of stroke survivors in the Amhara Region, Ethiopia (n=404).

Variable	Category	Frequency	Percentage
Comorbidity			
	Yes	257	63.6%
	No	147	36.4%
Number of falls			
	No	151	37.4%
	1	89	22.0%
	2	66	16.3%
	≥ 3	98	24.3%
Time for hospitalization			
	< 24 hours	307	76.0%
	> 24 hours	97	24.0%
Type of stroke			
	Hemorrhagic	158	39.1%
	Ischemic	246	60.9%
Duration of stroke			
	< 3 months	81	20.1%
	3–6 months	150	37.1%
	6–12 months	92	22.8%
	> 12 months	81	20.0%
Stroke complication			
	Yes	237	58.7%
	No	167	41.3%
Side of paralysis			
	Left	241	59.6%
	Right	163	40.4%
Duration of hospitalization			
	≤ 7 days	101	25.0%
	8–14 days	136	33.7%
	15–21 days	110	27.2%
	> 21 days	57	14.1%
Current medication use			
	Yes	235	58.2%
	No	169	41.8%
Loss of consciousness			
	Yes	226	55.9%
	No	178	44.1%
BMI			
	Underweight	28	6.9%
	Normal	237	58.7%
	Overweight	103	25.5%
	Obese	36	8.9%

n, sample size; BMI, body mass index; comorbidity includes hypertension, diabetes mellitus, cancer, and myocardial disorders.

### Magnitude of post-stroke depression and anxiety among the study subjects

**Figure 2:**
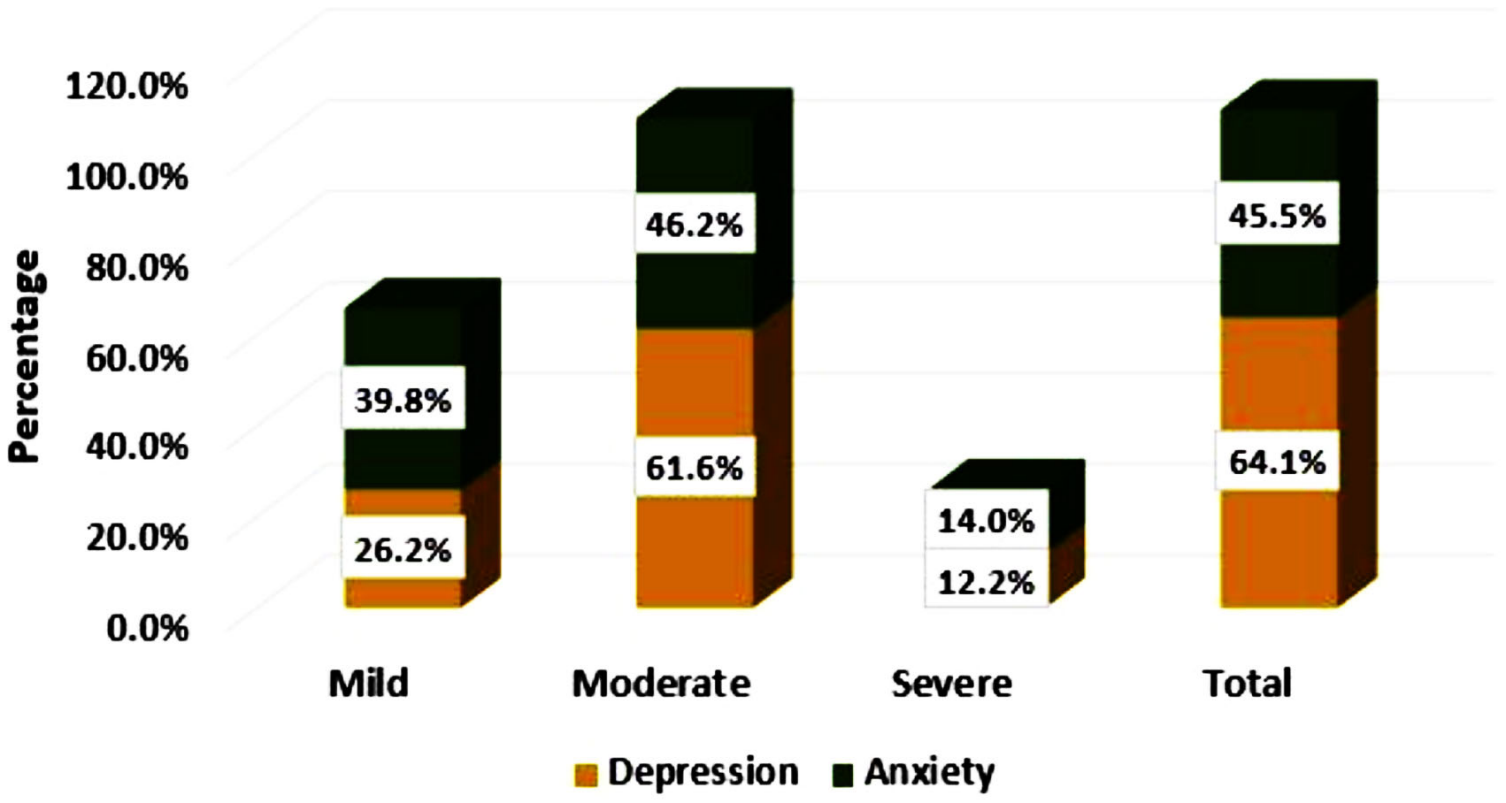
Levels of post-stroke depression and anxiety among stroke survivors in the Amhara Region, Ethiopia.

Of the total enrolled stroke survivors from the Amhara Regional State, 259 (64.1%) (95% CI: 59.3%–68.6%) developed clinically significant PSD. In contrast, 184 (45.5%) (95% CI: 40.7%–50.4%) study participants experienced PSA (**Figure 2**).

### Factors associated with post-stroke depression

Of all the variables considered, age, sex, educational status, occupation, drug abuse, stroke complication, duration of hospitalization, duration of stroke, current medication use, number of falls, and the presence of comorbidity were fitted to a multivariate binary logistic regression model as they yielded a p-value of less than 0.2 in the bivariate binary logistic regression model. Accordingly, the final multivariate binary logistic regression identified sex, stroke complication, and comorbidity as significantly associated with the occurrence of PSD (**Table 3**).

**Table 3:** Factors associated with post-stroke depression among stroke survivors in the Amhara Region, Ethiopia (n=404).

Variable	PSD		COR (95%CI) AOR (95%CI)	
	Yes(259) No(145)			
Age (in years)				
20–39	27	9	1.00	1.00
40–64	130	70	0.62 (0.27–1.39)	0.53 (0.20–1.46)
> 64	102	66	0.51 (0.23–1.16)	0.44 (0.16–1.26)
Sex				
Male	147	58	1.97 (1.30–2.97)	1.97 (1.06–3.67)*
Female	112	87	1.00	1.00
Educational status				
No formal education	78	42	1.01 (0.56–1.81)	1.56 (0.51–4.71)
Primary education	54	45	0.65 (0.36–1.19)	0.88 (0.30–2.62)
Secondary education	72	28	1.40 (0.75–2.61)	1.31 (0.46–3.77)
College and above	55	30	1.00	1.00
Occupation				
Farmer	22	19	1.00	1.00
Civil servant	36	21	1.48 (0.65–3.35)	1.01 (0.23–3.34)
Housewife	65	44	1.27 (0.62–2.63)	1.68 (0.63–4.51)
Merchant	51	25	1.76 (0.81–3.83)	1.79 (0.60–5.27)
Retired	18	11	1.41 (0.54–3.72)	2.85 (0.76–6.43)
Private	60	21	2.46 (1.12–5.43)	2.22 (0.74–6.64)
Other	7	4	1.51 (0.38–4.66)	2.33 (0.36–7.19)
Cigarette smoking				
Yes	91	32	1.91 (1.19–3.05)	1.12 (0.57–2.20)
No	168	113	1.00	1.00
Stroke complication				
Yes	171	66	2.32 (1.53–3.52)	2.83 (1.64–4.88)**
No	88	79	1.00	1.00
Duration of hospitalization				
≤ 7 days	71	30	1.85 (0.94–3.63)	1.40 (0.58–3.34)
8–14 days	79	57	1.08 (0.58–2.02)	0.67 (0.31–1.50)
15–21 days	77	33	1.82 (0.94–3.53)	0.89 (0.37–2.16)
> 21 days	32	25	1.00	1.00
Duration of stroke				
< 3 months	48	33	1.00	1.00
3–6 months	96	54	1.22 (0.70–2.13)	0.81 (0.39–1.70)
6–12 months	58	34	1.17 (0.63–2.16)	0.76 (0.32–1.78)
> 12 months	57	24	1.63 (0.85–3.13)	1.62 (0.66–3.97)
Current medication use				
Yes	157	78	1.32 (0.87–1.99)	0.80 (0.45–1.43)
No	102	67	1.00	1.00
Number of falls				
No	100	51	1.00	1.00
1 time	59	30	1.01 (0.57– 1.74)	0.70 (0.32– 1.51)
2 times	46	20	1.17 (0.63– 2.19)	0.67 (0.29– 1.54)
≥ 3 times	54	44	0.63 (0.37– 1.35)	0.42 (0.22– 1.04)
Comorbidity				
Yes	200	57	5.23 (3.36–7.14)	6.23 (3.91–9.19)**
No	59	88	1.00	1.00

n, sample size; PSD, post-stroke depression; *, P-value of< 0.05; **, P-value of< 0.001; Comorbidity includes hypertension, diabetes mellitus, cancer, and myocardial disorders.

This empirical study confirmed that the odds of PSD occurrence were almost twofold higher in men than their counterparts [adjusted odds ratio (AOR)=1.97, 95% CI: 1.06–3.67]. Furthermore, the manifestation of stroke-related complications increased the odds of PSD development by almost threefold compared to stroke survivors without any confirmed stroke complications (AOR = 2.83, 95% CI: 1.64–4.88). Furthermore, the presence of other systemic diseases increased the PSD occurrence (AOR=6.23, 95% CI: 3.91–9.19).

### Factors associated with post-stroke anxiety

First, variables such as educational status, occupation, average monthly income, time for hospitalization, side of the body affected by the stroke, duration of hospitalization, number of falls, comorbidity, and BMI passed the bivariate binary logistic regression and were fitted to the multivariate binary logistic regression model. Then, time for occupation, hospitalization, and the occurrence of other systemic comorbidities were found to be significantly associated with PSA among stroke survivors in the Amhara Regional State, Ethiopia (**Table 4**).

**Table 4:** Factors associated with post-stroke anxiety among stroke survivors in Amhara region, Ethiopia (n=404).

Variable	PSA		COR (95%CI) AOR (95%CI)	
	Yes (184) and No (220)			
Educational status				
No formal education	60	60	1.00	1.00
Primary education	38	61	0.62 (0.36–1.07)	1.58 (0.62–3.01)
Secondary education	48	52	0.92 (0.54–1.57)	1.48 (0.54–3.10)
College and above	38	47	0.81 (0.46–1.41)	2.21 (0.70–4.41)
Occupation				
Farmer	14	27	1.00	1.00
Civil servant	24	33	1.40 (0.61–3.22)	1.01 (0.28–3.06)
Housewife	55	54	1.96 (0.93–4.14)	1.72 (0.73–4.07)
Merchant	35	41	1.64 (0.75–3.62)	1.55 (0.58–4.14)
Retired	16	13	2.37 (0.89–4.29)	1.64 (1.91–4.72)*
Private	36	45	1.54 (0.71–3.36)	1.59 (0.59–4.26)
Other	4	7	1.11 (0.27–4.41)	1.55 (0.29–5.89)
Average monthly income (ETB)				
100–2,000	54	52	2.37 (0.89–4.29)	1.20 (0.51–2.84)
2,001–5,000	48	80	1.54 (0.71–3.36)	0.65 (0.31–1.41)
5,001–10,000	43	54	1.11 (0.27–4.41)	0.74 (0.37–1.47)
10,001–40,000	39	34	1.00	1.00
Time for hospitalization				
< 24 hours	148	159	1.57 (0.98–2.52)	2.05 (1.09–3.84)*
> 24 hours	36	61	1.00	1.00
Side of paralysis				
Left	110	131	1.01 (0.67–1.50)	1.09 (0.67–1.78)
Right	74	89	1.00	1.00
Duration of hospitalization				
≤ 7 days	55	46	2.04 (0.91–3.91)	2.18 (0.94–4.64)
8–14 days	62	74	1.43 (0.76–2.71)	1.13 (0.54–2.36)
15–21 days	46	64	1.23 (0.63–2.37)	0.89 (0.41–1.94)
> 21 days	21	36	1.00	1.00
Number of falls				
No	74	77	1.00	1.00
1 time	43	46	0.97 (0.57– 1.64)	0.85 (0.46– 1.60)
2 times	26	40	0.67 (0.37– 1.22)	0.52 (0.26– 1.06)
≥ 3 times	41	57	0.74 (0.45– 1.25)	0.75 (0.42– 1.34)
Comorbidity				
Yes	131	126	1.84 (1.22–2.79)	2.09 (1.32–3.29)**
No	53	94	1.00	1.00
BMI				
Underweight	10	18	1.00	1.00
Normal	110	127	1.56 (0.69–3.52)	1.58 (0.62–4.02)
Overweight	44	59	1.34 (0.56–3.19)	1.48 (0.54–4.10)
Obese	20	16	2.25 (0.81–6.21)	2.21 (0.71–6.93)

n, sample size; PSA, post-stroke anxiety; ETB, Ethiopian birr; *, P-value of 0.05-0.01; **, P-value of< 0.01; BMI, body mass index; Comorbidity includes hypertension, diabetes mellitus, cancer, and myocardial disorders.

As compared to farmers, retired stroke survivors were 1.64 times more likely to have PSA (AOR=1.64, 95% CI: (1.91–4.72). Time of hospitalization was also significantly associated with PSA. Individuals who visited the hospital within a day after the stroke occurred were two times more likely to have anxiety than late presenters (AOR = 2.05, 95% CI: 1.09–3.84). Similarly, PSA was two times more likely to occur in stroke survivors with a systemic comorbidity (AOR=2.09, 95% CI: 1.32–3.29).

## Discussion

Common mental disorders such as anxiety and depression, along with infectious diseases, create a double burden and serious public health issues, particularly in developing countries. Understanding the actual burden on different segments of the community could be used as baseline data to inform policymakers, leading to modified, efficient, and evidence-based clinical practice and resulting in improved quality of life for stroke survivors. Although the cross-sectional nature of the study design hindered the assessment of causality and potential social desirability bias associated with subjective response was a challenge, an empirical multicenter study assessed the burden of PSD and PSA and their predictors among stroke survivors.

The prevalence of PSD among stroke survivors in the Amhara Regional State was found to be 64.1%. This figure denotes that almost two-thirds of the stroke survivors in the Amhara Regional State are depressed, implying the advantage of integrating basic screening and monitoring of common mental disorders in the routine checkups and rehabilitative therapy of stroke survivors (24, 41). The burden of depression revealed by this study was in line with a report from India (42).

This burden was lower than studies from India (73.1% and 90%) (23, 43) and a report from Korea (70%) (24). The socioeconomic and study population variations could have contributed to such significant differences in prevalence (23) as there were fewer study participants enrolled in the Indian studies. Furthermore, the second study was conducted on relatively older and rural communities (23). Moreover, more than 90% of the stroke survivors in one of the Indian studies (23) were living with the stroke for more than a year which could create an opportunity for stroke-related complications and disabilities resulting in an aggravated and inflated prevalence of depression (44, 45).

In contrast, the prevalence reported in this study was higher than all similar studies conducted in Ethiopia (27.5%–49.6%) (4, 25, 26) and studies conducted worldwide (14%–57.1%) (22, 45–49). This could be potentially due to socioeconomic, methodological, temporal, and spatial variations. As it was supported by multiple studies (50–52), a study investigated the global temporal pattern of depression, revealing a rising burden, especially in sub-Saharan Africa, Middle-East, and Far East Asia (53). Furthermore, the variation in the tools used to assess PSD was also a possible reason behind this discrepancy. PSD was evaluated in this investigation using the PHQ-9 questionnaire, a brief, readily adaptable, and reasonably effective tool for chronic patients that may have overstated the prevalence of PSD (54, 55). Continuous and progressive assessment and intervention are advised to address the burden of depression.

Consistent with evidence from (24), the prevalence of PSA was observed in almost half, 45.5%, of stroke survivors living in the Amhara Regional State. Consistent with other studies, the prevalence of PSD in this study was higher than PSA. However, we have not found any study that investigated the anxiety burden in stroke survivors in Ethiopia. PSA was confirmed to be more prevalent than the prior burden abroad, which ranged from 15.7% to 38.3% (1, 30, 56–58). This inconsistency could also be associated with socioeconomic factors, the study population, and methodological differences (56). An advanced and integrated healthcare service with better healthcare coverage in China potentially reduces the prevalence of PSA among stroke survivors in China (57). Whereas, a fragile healthcare system aggravated by the poor socioeconomic status of the Ethiopian population could inflate the PSA burden on Ethiopian patients. Systematic review and meta-analyses assessed pragmatic studies carried out in Europe. The European nations have strong healthcare systems that enable effective management and control of stroke as well as a better average annual income, causing stroke survivors to anticipate a healthy future and be optimistic. This could contribute to the relatively lessened prevalence of PSA in the European population (59). The significant negative impact of these prevalent common mental disorders on the prognosis of rehabilitative care, quality of life, and daily activity of stroke survivors requires stakeholders to act quickly (60). We suggest studying the burden of PSA in different segments of stroke survivors in Ethiopia and other third-world nations.

In the Amhara Regional State, male stroke survivors were twofold more likely to experience PSD than their counterparts. In the majority of Ethiopian families, men are the breadwinners of families, putting them in charge of family-related financial expenses (46). After the occurrence of a stroke, those responsible individuals could face an economic challenge, leading them to be more depressed. This was supported by research from a study conducted in Gondar, Ethiopia, and two international studies (44, 61, 62), but was not supported by other results from other studies (4, 22, 42). The probable reasons cited for the more prevalent PSD in other studies were genetic predisposition and sex-based differences related to fewer women in rehabilitative care (42).

Consistent with evidence from India (22, 46), the PSD occurrence was statistically significantly higher in stroke survivors suffering from complicated stroke. Since the occurrence of complications is highly determined by the duration, recurrence, severity, and comorbidity of the stroke, these conditions could aggravate the occurrence and severity of depression by making the patients desperate, disabled, and socioeconomically deprived (23, 46).

Stroke occurrence in accordance with other systemic diseases increased the PSD burden among stroke survivors in the Amhara Regional State. This association was also observed in study participants of research conducted in India (45). The direct and indirect costs that needed to be paid and follow-up-related challenges could be the reasons behind this relationship.

It is possible that, due to the more anxious personal behavior of the participants, stroke survivors hospitalized within a day after the stroke onset had more anxiety than those who reached healthcare centers at a later time. As compared to farmers, retired participants were likely to experience PSA. This is because retired individuals are mostly old, and retirement by itself is a possible cause of retirement-related stress, possibly aggravating and inflating the occurrence of PSA among retired stroke survivors (63). Similar to depression and consistent with evidence from India (64), study subjects who suffered from additional systemic disorders, such as diabetes mellitus, hypertension, and other cardiovascular conditions, were more likely to develop PSA.

## Limitations of the study

The cross-sectional design we used restricts the ability to infer causality between the independent and dependent variables, emphasizing the need for longitudinal research to understand how these factors interact over time. Additionally, study participants might underreport their drug addiction behaviors and economic-related information, which could expose the output of this study to social desirability bias. We have used a variety of strategies to overcome such potential biases that could affect the output. Detailed explanations of the threat caused by their misinformation and our strong measure of privacy and confidentiality of their response and data were among the basics. We advise objective assessment of exposure to drugs in future research. Furthermore, the potential association of variables with depression is better assessed qualitatively in their natural living area. This creates an opportunity to explore conditional, social, and environmental factors with potential associations with common mental disorders among stroke survivors.

## Conclusion

Among the stroke survivors who presented to physiotherapy clinics of CSHs in the Amhara Region, a relatively high prevalence of mental disorders was observed. Almost two in three stroke survivors experienced PSD whereas around half were suffering from PSA.

When considering associated factors, variables such as sex, stroke complication, and comorbidity showed significant associations with the occurrence of PSD. Furthermore, retirement, time for hospitalization, and comorbidity were significant predictors of PSA among stroke survivors attending CSHs in the Amhara Region, Ethiopia. Promoting mental health care in accordance with routine rehabilitative therapy for stroke patients helps in patient management and makes the endeavor successful. Early mental health screening and diagnosis of double-burdened segments of the population, such as those of old age, complicated case patients, and retired stroke survivors, will help healthcare providers anticipate early interventions, including favorable coping styles.

## Data Availability

The original contributions presented in the study are included in the article/supplementary material. Further inquiries can be directed to the corresponding author.
